# Electronic Cigarette Use among Irish Youth: A Cross Sectional Study of Prevalence and Associated Factors

**DOI:** 10.1371/journal.pone.0126419

**Published:** 2015-05-27

**Authors:** Kate Babineau, Keishia Taylor, Luke Clancy

**Affiliations:** TobaccoFree Research Institute Ireland, DIT Focas, Camden Row, Dublin, Ireland; Legacy, Schroeder Institute for Tobacco Research and Policy Studies, UNITED STATES

## Abstract

**Purpose:**

To examine prevalence of, and factors associated with, e-cigarette use among young people aged 16-17 in Ireland.

**Methods:**

In 2014, a representative sample of 821 young people aged 16-17 recruited from secondary schools completed a pen and paper survey on e-cigarette use, tobacco use, and socio-demographic items.

**Findings:**

A total of 23.8% of respondents had used e-cigarettes at least once. Dual trial of tobacco and e-cigarettes was common with 69.5% of regular smokers and 30.4% of ‘ever’ smokers having tried e-cigarettes and 10.6% of current smokers using e-cigarettes regularly. 4.2% of never smokers have tried e-cigarettes. Overall, current e-cigarette use (once a month or more) was low (3.2%). Binary logistic regression conducted through generalized estimating equations (GEE) determined that controlling for other variables, current tobacco use and ‘ever’ tobacco use predicted ever e-cigarette use. Gender and school-level socioeconomic status were also independent predictors of ever e-cigarette use. Gender stood as the only predictor of on-going e-cigarette use, with males being more likely to regularly use e-cigarettes at least once a month.

**Conclusions:**

E-cigarette use among 16-17 year olds in Ireland is of note, with nearly a quarter of students having tried them. Concurrent or experimental use of e-cigarettes and tobacco is more common than sole use, while a small number have tried e-cigarettes without having tried tobacco.

## Background

Electronic cigarettes (e-cigarettes) and other battery-operated nicotine delivery devices appeared in the European and US markets less than a decade ago. Over the past ten years, they have developed into a billion dollar industry [1].As the industry has grown, so has the public debate surrounding the products. Physicians, tobacco control activists, policy-makers, and healthcare professionals weigh in on the discussion, with those from all backgrounds adopting both for- and against- positions. Those in favor of e-cigarettes often adopt a harm reduction stance, arguing that they can lead to lessening or cessation of tobacco use among individual smokers and decrease exposure to second hand smoke for non-smokers. Many e-cigarette users report that they begin using the product as a cessation aid, though the efficacy of this approach remains unclear [2–3]. Others argue that e-cigarettes purport to be a harmless cessation tool when in fact, they may delay quitting, promote dual use, or serve as a ‘gateway’ to tobacco use [1][4].Furthermore, they threaten to undermine the progress made by Tobacco Free laws by renormalizing the act of smoking in public places and may actually negatively impact air quality, expanding the public health risk to those who are not directly engaging with the product [5–6].This public debate regarding the potential benefits, risks, and efficacy of e-cigarettes is on-going and unlikely to be resolved, concretely, in the near future. There are an increasing number of empirical studies on the topic, though the need for more research to contribute to the collective discussion is imperative as policies on e-cigarettes are still in the crucial, formative stage.

### E-Cigarette Prevalence among Young People

There are a small yet increasing number of studies on e-cigarette use among young people. In the US, the National Youth Tobacco Survey (NYTS) found that e-cigarette use among teenagers increased dramatically from 2011–2012, with ever use among rising from 4.7% to 10.0%, while the Centre for Disease Control and Prevention (CDC) reported that 9.3% of high school students who had never smoked tobacco had tried used e-cigarettes [7–8]. These high figures have been contested, with others reporting that less than 1% of adolescent never smokers in the US had tried e-cigarettes [9]. In Canada, a reported 16.1% of young people aged 16–30 have tried e-cigarettes, including 5.1% of non-smokers [10]. Gender, socioeconomic status, and personal tobacco use have all been found to be predictors of e-cigarette use.

In the European context, research suggests that e-cigarette use is increasing, particularly among tobacco smokers. In 2012, the Special Eurobarometer 385 surveyed young people and adults over the age of 15 in 27 EU countries and found that 20.3% of smokers had used e-cigarettes, and of those, 9.0% use them regularly [11]. However, 1.1% of those who have never smoked had tried e-cigarettes. In Eastern Europe, where there is a documented history of higher- than-average tobacco use among youth, there are also higher reported rates of e-cigarette use. In Poland, a 2010–2011 study reported 23.5% of 15–19 had tried an e-cigarette and 8.2% used the previous 30 days [12]. High rates of dual use were also reported, with tobacco smokers using e-cigarettes more frequently than non-smokers. E-cigarette use is also high among Hungarian (13.0%) and Lithuanian (9.1%) youth [13]. In the UK, the Action on Health and Smoking (ASH) reported notably lower levels among adolescents: 8% tried them once or twice and 2% used them regularly [14]. Among non-smoking adolescents, only 1% had tried them once or twice [15].

As evidenced, the available research on young people and e-cigarettes is disparate. Reported prevalence varies greatly from 2% to 12% in the US, from 1% in the UK to 23.5% in Poland. In Ireland, there is no available data on young people and e-cigarette prevalence. The wide discrepancies in reported use, the potentially harmful implications of e-cigarette initiation (gateway, dual use), and the general lack of available information on adolescents’ relationship with e-cigarettes underscore the immediate need for emerging research on the topic. The current study uses data from the 2014 Youth Perception of Plain Packaging Study, a nationally representative school-based survey of 16–17 year olds in Ireland to explore: a) the awareness of e-cigarettes; b) the prevalence of e-cigarette use among the population and sub-groups; and c) predictive factors associated with e-cigarette use.

## Methodology

### Recruitment Strategy

Young people in their fifth year of secondary school, aged 16–17, were chosen as the target population as this is an age with a high level of smoking initiation among young people in Ireland. Also, students in this year of schooling are more accessible than the students one year immediately below and above them due to the structure of the Irish secondary school system. A representative sample of secondary schools from around the country was selected for participation. The sample of schools was stratified on the basis of several factors including: a) geographic location, b) school size, c) type of school (boys, girls, co-ed), d) religious affiliation (Catholic, Church of Ireland, inter-denominational), and e) school-level socioeconomic status (schools designated ‘disadvantaged’ by the state vs. non-disadvantaged schools). After stratification according to the sampling criteria, individual schools were randomly selected for inclusion. Overall, fifth year students from 16 secondary schools across Ireland were asked to participate.

### Survey Administration

School principals were initially contacted with a written letter asking for their support in conducting this research. These letters were followed with phone calls a few days later, explaining the research process and the protocol for participation. After arranging a time with the principal and participating teachers, a researcher travelled to the school to administer the questionnaire. To facilitate the individual needs of each school, researchers adopted a flexible approach to survey administration. Surveys were administered in a ‘pen and paper’ format. Data collection occurred from March-June 2014.

### Ethical Issues

When conducting research with young people, there are a number of ethical considerations to take on board. Prior to administering the survey, information sheets and consent forms were distributed to all students and parents in participating schools. Active consent was received from all participating students. Parental consent was obtained through an ‘opt out’ method, meaning that parents could give ‘non-consent’ to their children taking part in the research. All students were informed that the research was voluntary, anonymous, and if students posed any questions, they were answered honestly and directly by researchers present. They were also informed that this was not a test and there were no ‘right’ or ‘wrong’ answers. The study received ethical approval from Dublin Institute of Technology’s Research Ethics Board.

### Sample

A sample of 901 young people from 16 secondary schools were administered a questionnaire. Out of this group, five participants opted out of participation and an additional 75 had missing data for some of the key variables in this analysis and were omitted. This left us with a final sample of 821. The average age of participants was 16.6 years old. There was a near equal gender divide, with 49.8% male and 50.2% female. A total of 592 (71.1%) attended non-disadvantaged schools with 229 (27.9%) attending designated socioeconomically disadvantaged schools. The majority of students were born in Ireland or in the UK (685, 83.4%), with an additional 63 (7.7%) from Eastern Europe, and 73 (8.9%) born elsewhere. The final sample was representative of the national figures for secondary school students with regards to gender and DEIS enrollment and thus, weighting of data for this particular analysis was deemed unnecessary.

### Measures

A questionnaire was constructed for the study drawing on existing, validated tobacco items recommended by the World Health Organization and the Center for Disease Control and Prevention. The current measure was piloted and tested in two secondary schools in the Irish context to ensure that question format, wording, and layout were straight-forward, age appropriate, and conducive to data collection. Minor changes were made to the questionnaire prior to full-scale implementation.

#### E-Cigarette Awareness / Prevalence

All respondents were asked ‘*have you ever heard of e-cigarettes*?’ with yes/no response categories. Respondents were then asked to select one of the following statements that best describes them: (*I have never tried an e-cigarette*, *I have tried an e-cigarette once or twice but don’t use one regularly*, *I used to smoke e-cigarettes but have given up*, *I smoke an e-cigarette at least once a month*, *I smoke an e-cigarette at least once a week*, *I smoke an e-cigarette every day*). These items were adapted from the smoking prevalence item recommended by the WHO and the CDC, replacing the word ‘cigarette’ with ‘e-cigarette’. For this analysis, participants were dummy coded into two categories: Ever Users (those who had tried e-cigarettes) and Never Users (those who had never tried e-cigarettes).

#### Personal and Family Tobacco Use

Participants were asked if they had ever smoked a cigarette and if currently smoking, how frequently (*everyday*, *at least once a week*, *at least once a month*, *tried smoking once or twice but don’t smoke now*, *used to smoke but quit*, *never smoked*). Responses were recoded into ‘Current Smokers’ (those who smoke at least once a month), ‘Ever Smokers’ (those who have tried smoking once or twice or have quit), and ‘Never Smokers’ (those who have never tried cigarettes). Participants were also asked about the smoking habits of their immediate family members. A dichotomous variable was created to distinguish between those who had an immediate family member who smoked (mother, father, siblings) and those who did not.

#### Socio-demographic Information

Socio-demographic variables included gender, country of birth, and school-level socioeconomic status. Country of birth was divided into three categories: 1) Ireland/UK, 2) Eastern Europe, and 3) Elsewhere. This categorization was derived on high reported levels of e-cigarette use among Eastern Europeans in recent studies. Given the relatively large number of Eastern European students enrolled in Irish schools, it was decided to explore this possible association in the Irish context [16]. School level socioeconomic status was measured using a dichotomized variable: students attending a socioeconomically disadvantaged school (as designated by the state) and those not [17].

### Analysis

Frequencies were calculated for e-cigarette use for all participants and χ2 were performed to examine significant differences between sub-groups in the sample. E-cigarette use was assessed as a function of demographic and tobacco-related characteristics using Generalized Estimating Equations (GEE) [18]. GEE provides a framework for the analysis of non-normal correlated data with binary outcomes and due to the classroom sampling strategy in the current study, there is an increased likelihood of correlated data in our sample. While multi-level or mixed-effect models have become popular in the social sciences, GEE models also provide a framework for the analysis of non-normal correlated data [19–20]. The GEE approach has been proven effective and appropriate for this type of analysis, wherein there is intra-cluster dependence and unbalanced clusters of small numbers [21–23]. For our analyses, we employed an exchangeable working correlation matrix structure with a binomial probability distribution and a logit link function. Both individuals and classrooms were entered as subject variables.

Five independent variables were selected for inclusion in the GEE binary logistic regression model to predict e-cigarette use: a) gender, b) school level SES, c) region of birth, d) tobacco use in the immediate family, and e) personal tobacco use. These variables were selected because they have been determined to be predictors of e-cigarette use in other studies (gender, personal tobacco use), and because they are widely recognized predictors of tobacco use (tobacco use in family, country of birth, socio-economic disadvantage). Interaction effects between predictor variables were investigated. E-cigarette use was analyzed through a binary variable (those who had ever smoked e-cigarettes and those who never smoked e-cigarettes), with those who were unaware of e-cigarettes or did not respond coded as missing and removed from analysis. This left a total of 792 (88.2%) of the sample for inclusion in the regression analyses. Following the initial analysis, an additional layer of analysis was conducted to explore predictors of continued e-cigarette use among ‘ever users’. We removed ‘never’ e-cigarette users from the model and ran a GEE binary regression with the predictor variables listed above to explore predictors of regular e-cigarette use compared with ‘ever’ use. There were 196 participants included in this layer of analyses. All tests were performed using SPSS, Version 21 (IBM, Illinois).

## Findings

### Prevalence

Table 1 presents the descriptive findings of the sample. 18.4% (151) were current tobacco smokers, 29.2% (240) were ever smokers, and 52.4% (430) have never smoked tobacco.

**Table 1:** Descriptives of the sample.

Demographic Variable	Response Categories	Frequency	Valid Percentage
**Gender**	Male	409	49.8
	Female	412	50.2
**Socioeconomic status**	Attending disadvantaged school	229	28.1
	Attending a non-disadvantaged school	592	71.9
**Birth Region**	Ireland / UK	685	83.6
	Eastern European Country	63	7.8
	Elsewhere	73	8.6
**Personal Tobacco Use**	Current Smoker	151	18.4
	Ever Smoker	240	29.2
	Never Smoker	430	52.4
**Family Tobacco Use**	Smoker in immediate family	384	46.8
	No smoker in immediate family	437	53.2
**E-Cigarette Use**	Ever smoked e-cigarettes	196	24.0
	Never smoked e-cigarettes	625	76.0

The majority of participants (89.0%, 797) were aware of e-cigarettes. A total of 196 participants (24.0%) had used e-cigarettes at least once and 26 participants (3.2%) were current users (at least once a month). Young men (106, 26.5%) were significantly more likely to have tried e-cigarettes than young women (88, 21.5%) [*X2* (2, n = 814) = 6.51, p<.05]. E-cigarette use was higher among students in designated disadvantaged schools (75, 32.6%) than non-disadvantaged schools (122, 20.6%). Differences also emerged along the lines of birth country, with young people born in Eastern Europe being significantly more likely to have tried e-cigarettes (28, 44.4%) than those born in Ireland / UK (150, 21,9%) or elsewhere (17, 23.6%).

Frequency data for e-cigarette use categorized by tobacco use shows the high percentage of smokers who have used e-cigarettes. As shown in Table 2, 69.5% of current tobacco smokers have tried e- cigarettes. Of ‘ever-smokers’, that figure drops to 30.4%. Among never smokers, the rate was 4.2%.

**Table 2:** E-cigarette use among young people, arranged by tobacco smoking status (N, %).

	Never Tobacco Smokers (N = 430)	Ever Tobacco Smokers (N = 240)	Current Tobacco Smokers (N = 151)	Total (N = 821)
**Never E-Cig Users**	95.8% (412)	69.6% (167)	30.5% (46)	76.1% (625)
**Ever E-Cig Users**	3.7% (16)	27.1% (65)	58.9% (89)	20.7% (170)
**Current E-Cig Users**	0.5% (2)	3.3% (8)	10.6% (16)	3.2% (26)

### Factors associated with e-cigarette use

First, socio-demographic determinants and predictive factors were evaluated using bivariate regression. Each of the five independent predictors was tested individually for inclusion in a binomial logistic regression model. Through these preliminary analyses, three factors were significantly associated with e-cigarette use: attending a socioeconomically disadvantaged school, being born in an Eastern European country or elsewhere when compared with being born in Ireland or the UK, and being a ‘current’ or ‘ever’ tobacco user compared with being a never user.

A GEE binary logistic regression was conducted to predict e-cigarette use including the significant predictors from the bivariate regressions. A test of the full model against a constant only model was statistically significant, indicating that the predictors as a set reliably distinguish between ever e-cigarette users and never e-cigarette users. Table 3 displays the results of the logistic regression analyses examining factors associated with e-cigarette use. Of the included demographic factors, gender and school-level socioeconomic status were significant predictors of e-cigarette use. Specifically, females were less likely to have tried e-cigarettes [OR = 0.73, 95% CI = (0.54–1.10)] while those who attended a disadvantaged school were more likely to have used e-cigarettes [OR = 1.77, 95% CI = (1.09–2.87)]. When controlling for demographic variables, personal tobacco use was significantly associated with e-cigarette use. Current smokers [OR = 54.85, 95% CI = (29.94–100.49)] were much more likely to have used e-cigarettes than ever smokers [OR = 11.20, 95% CI = (6.37–19.07)] and non-smokers. No interactions between independent variables were significantly associated with current use of e-cigarettes.

**Table 3:** GEE Model of predictive factors of e-cigarette use.

	Coefficient	OR (95% CI)	Significance
**Female**	-0.42	0.73 (0.54–1.10)	.05*
**Socioeconomically disadvantaged school**	.57	1.77 (1.09–2.87)	.02*
**Born in Eastern Europe**	.24	1.27 (0.64–2.52)	.49
**Born Elsewhere**	.44	1.56 (0.79–3.08)	.20
**‘Ever’ Tobacco User**	2.41	11.20 (6.37–19.07)	p<.001**
**Current Tobacco User**	4.00	54.85 (29.94–100.49)	p<.001**

Comparison groups are: Male, Non-disadvantaged school, born in Ireland/UK, Smoker in Family, non-tobacco user

*Statistically significant at p<.05

**Statistically significant at p<.001

### Predictors of continued e-cigarette use

An additional layer of analysis was conducted to explore factors associated with continued e-cigarette use. For this analysis, the never e-cigarette users were removed from the model and predictive factors were tested to determine what may influence those who use e-cigarettes on an on-going basis [ever users (n = 170) vs. current users (n = 26)]. A test of the full model against a constant only model was statistically significant, indicating that the predictors as a set reliably distinguish between ever e-cigarette users and regular e-cigarette users. Table 4 displays the results of the GEE analysis examining associated factors. Of the included demographic predictors and smoking status, gender stands alone as the sole predictor of current e-cigarette use, with females being less likely than males to continue to use e-cigarettes on a regular basis [OR = 0.38, 95% CI = (0.16–0.94)].

**Table 4:** GEE Model of predictive factors of continued e-cigarette use.

	Coefficient	OR (95% CI)	Significance
**Female**	-.96	.38 (0.16–0.94)	.04*
**Socioeconomically disadvantaged school**	-.28	.76 (0.32–1.82)	.54
**Born in Eastern Europe**	.51	1.67 (0.51–5.45)	.40
**Born Elsewhere**	.83	2.28 (0.69–7.60)	.18
**‘Ever’ Tobacco User**	-.40	.96 (0.18–5.14)	.96
**Current Tobacco User**	.42	1.53 (0.30–1.82)	.61

Comparison groups are: Male, Non disadvantaged school, born in Ireland/UK, Smoker in Family, non-tobacco user

*Statistically significant at p<.05

## Discussion

This paper presents findings from the first Irish study to explore e-cigarette prevalence among a nationally representative sample of young people. E-cigarette use among 16–17 year olds in Ireland is relatively high (24.0%), with figures similar to those reported in Eastern Europe and some US studies [7][12]. Young people who smoke tobacco are significantly more likely to have tried e-cigarettes than those who do not smoke. However, there a small number of young people who had never tried tobacco had also tried e-cigarettes (4.2%). This figure is more than three times that reported by ASH in the UK (1.1%) but less than half of that reported by the CDC in the USA (9.8%) [8][14].

This study also explored social determinants and smoking behaviour as possible predictors of e-cigarette use based on international literature and established predictors of tobacco use. In line with studies in the US, Canada, and Poland, gender was found to be a predictor of e-cigarette use in Ireland, with males being more likely to have tried the product [10][12][24]. School-level socioeconomic status was also a predictor, with those attending ‘disadvantaged’ schools being more likely to have used e-cigarettes. National studies conducted in Poland, Lithuania, and Hungary indicated high levels of tobacco and e-cigarette use in these locations [11–12].^.^ In our sample, however, birth country did not prove to be a significant predictor in the regression model. This could indicate that country of residence inclusive of peer networks and social norms, rather than country of birth, is related to e-cigarette use. In line with nearly all of the existing research on e-cigarettes and young people, tobacco use emerged as the largest predictor of e-cigarette use. Current tobacco users were much more likely to have tried e-cigarettes than non-smokers or ever smokers [25–26]. However, tobacco users were not significantly more likely to continue to use e-cigarettes regularly. When comparing those who have *tried* e-cigarettes with those who currently use e-cigarettes on a regular basis, gender emerged as the sole predictive factor. Young males were significantly more likely to use e-cigarettes at least once a month than their female counterparts. This corresponds with a recent review of three USA-based studies on youth and e-cigarettes, two of which found gender to be the strongest predictor of on-going e-cigarette use [27].

The main limitations of this study are: as a cross-sectional study, the data is only representative of young people in Ireland at a given point. A longitudinal quantitative study would be able to provide more nuanced information about the progression of e-cigarette use over time. From a methodological standpoint, conducting research in schools with a pen and paper survey has benefits and shortcomings. For example, we had access to a representative sample but some participants did not complete the questionnaire or skipped key questions, resulting in data that had to be removed from analyses. Also, the questionnaire was limited with regards to motivations for, and the nature of, e-cigarette use. Additional questions on the context of first use and intentions for on-going use could have provided a better-rounded illustration of the subject matter. Further, the total numbers for the regression analysis on ever e-cigarette use vs. current e-cigarette use were low. This test could have benefited from a larger sample size.

Overall, these findings provide a grounding from which inquiry on e-cigarette use among young can continue to develop. The data shows that e-cigarettes are widely tried by young people in Ireland, particularly by those who smoke or have tried smoking. Irish data aligns with international data, demonstrating a relationship between tobacco use and e-cigarette use. However, regular tobacco use is not a predictor of regular e-cigarette use, suggesting that on-going dual use of both products is not as common as experimentation. The data does not lay rest to the ongoing debate surrounding e-cigarettes among healthcare professionals, policy makers, and the media. In many ways, these findings may raise additional questions and points of interest. However, this report provides valuable, representative data about e-cigarette use among young people in Ireland and begins to explore social determinants and predictive factors. Further research is needed to explore young people’s motivations for use, intentions for further use, access to e-cigarettes, and the nature of ‘dual use’ of tobacco and e-cigarettes (i.e. a tool for quitting, concurrent use, experimentation).
